# Parapharyngeal and maxillary metastasis in hepatocellular carcinoma as the first presentation: a rare case

**DOI:** 10.1016/j.bjorl.2024.101459

**Published:** 2024-06-13

**Authors:** Shan Chen, Hua Cai, Bei-Bei Gao, Wen-Wen Wang

**Affiliations:** a Huazhong University of Science and Technology, Tongji Medical College, Union Hospital, Department of Otorhinolaryngology, Wuhan, China; b Huazhong University of Science and Technology, Tongji Medical College, Union Hospital, Department of Pathology, Wuhan, China

## Introduction

Hepatocellular Carcinoma (HCC), also known as hepatoma, is the most common type of primary liver cancer. HCC originates from hepatocytes, which are the main cells of the liver, and it is a highly aggressive and potentially fatal malignancy. In China, approximately 466,000 new liver cancer cases and 422,000 liver cancer deaths occur each year, accounting for approximately 50% of liver cancer cases globally.^1^

The Parapharyngeal Space (PPS) is a triangular compartment bordered by the hyoid bone and skull base. The PPS is divided into pre- and retro-styloid compartments by the styloid process and the associated attached stylopharyngeal aponeurosis. PPS tumors are rare in the head and neck region, comprising only 0.5%–1% of all head and neck neoplasms. The majority of PPS tumors, including approximately 70 pathological types, are benign. Metastatic lesions are rarely found in the PPS, with the thyroid being the most common primary tumor site.^2^

In this report, we describe a case of parapharyngeal and maxillary metastases, revealing multi-metastatic HCC in an asymptomatic 64-year-old man.

## Case report

**Figure 1:**
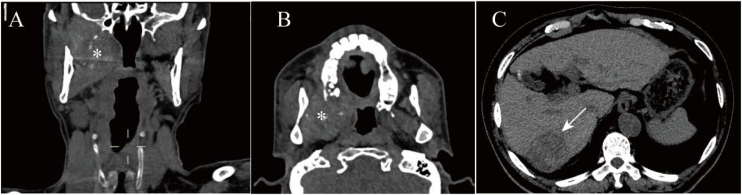
(A and B) A CT scan of the oropharynx revealed a soft tissue density mass on the right side of parapharyngeal space, involving the maxilla; (C) Abdominal CT scan showing a low-density mass with a distinct boundary in the right lobe of liver.

A 64-year-old male patient experiencing limited mouth opening and discomfort while swallowing was referred to the Department of Otorhinolaryngology, Head and Neck Surgery, in January 2021. Physical examination revealed that the right palatine tonsil to have been pushed medially. A Computed Tomography (CT) scan exhibited a soft-tissue mass in the right PPS, measuring 4 cm at its largest cross-section. The enhanced scan showed heterogeneous, moderate-to-intense enhancement of the mass, involving the pterygomaxillary fossa, infratemporal fossa, and the right lateral wall of the pharynx. In addition, bone destruction was noted in the posterior wall of the right maxillary sinus (Fig. 1A and B). The patient underwent surgical resection of the parapharyngeal tumor through a transcervical approach without mandibulotomy. However, severe bleeding occurred during the procedure, and a transoral approach was used to manage the bleeding. Subsequently, the patient also underwent tracheostomy, and the mass was partially resected, with bleeding controlled by local packing with iodoform gauze.

**Figure 2:**
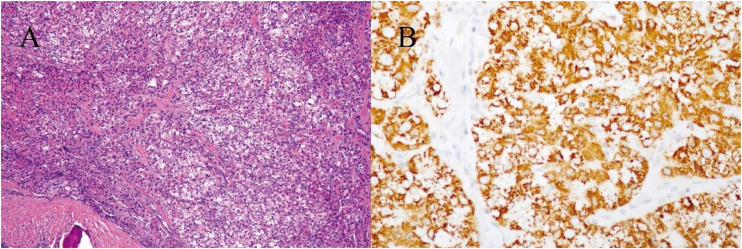
(A) Histological examination showing the presentation of poorly differentiated carcinoma. (B) The immunohistochemical result shows a strong expression of anti-hepatocyte-specific antigen in neoplastic cells and were diagnosed as indicative of HCC metastasis.

Histological analysis revealed a poorly differentiated carcinoma (Fig. 2). The immunohistochemical profile confirmed the presence of metastatic HCC, indicated by the following markers: positive for hepatocyte, AR, and CK8/18 and negative for CD34, HSP70, Syn, CgA, SOX 100, CK7, Alpha-Fetoprotein (AFP), CD56, P63, and mammaglobin. Further CT evaluation showed a mixed-signal mass in the posterior segment of the right liver lobe (Fig. 1C) and bone destruction in the right femoral neck, suggesting that the primary tumor originated in the liver and had metastasized to the femur.

The patient received radiation therapy targeting the parapharyngeal mass with a total dosage of 64 Gy delivered in 20 fractions over 11 sessions. Following radiation therapy, the patient underwent six cycles of immunotherapy with camrelizumab, each with a dose of 200 mg. In addition, liver-directed stereotactic body radiation therapy was administered to treat the liver lesion. One year ago, the patient experienced a femoral fracture and did not receive further treatment for it. Despite these complications, the patient is still alive at present.

## Discussion

HCC is the most common type of primary liver cancer and has the third highest mortality rate worldwide. Risk factors for HCC include liver cirrhosis, chronic hepatitis, alcohol consumption, nonalcoholic liver steatosis, and exposure to mycotoxins. The most common metastasis sites for liver cancer are the lungs, lymph nodes, bones, and brain. Metastasis to the maxilla and PPS is rare.^3^ AFP is the primary biomarker used for HCC screening. However, 30% of patients with liver cancer do not exhibit high AFP levels. Our patient had a history of alcohol abuse, but not viral hepatitis, and his AFP levels were within the normal range, making the diagnosis of metastatic HCC in the PPS particularly challenging.

Tumors in the PPS can originate there, extend directly from adjacent areas, or result from metastasis from regional or distant sites. Because of the deep location, complex anatomy, and critical structures within the PPS, preoperative cytological diagnosis using Fine-Needle Aspiration Biopsy (FNAB) is recommended for managing these tumors.^4^ FNAB is particularly advised for diagnostic purposes when HCC is suspected.^5^ However, in our patient, intractable hemorrhage occurred during the biopsy procedure, and it could not controlled through electrocauterization, suture ligation, or Surgicel alone. The bleeding was successfully stopped using a transoral approach involving packing with iodoform gauze, without the need for extensive procedures, such as external carotid artery ligation or embolization. The iodoform gauze was removed 3 days post-operation without a second episode of hemorrhage. Although the patient did not have coagulopathy, the vascular nature of the metastatic HCC lesion posed a life-threatening risk during biopsy. FNAB should be performed to diagnose PPS tumors and prevent severe hemorrhage.

Patients diagnosed with HCC and bone metastasis often have a poor prognosis and experience various skeletal-related complications, impaired mobility, decreased quality of life, increased medical costs, and shortened overall survival time. Our patient did not undergo surgical treatment for HCC due to the presence of multiple metastases. Although the patient experienced a femoral neck fracture and discontinued treatment, disease control and prolonged survival were achieved through the administration of camrelizumab and radiotherapy. This finding suggests that immunotherapy, particularly using inhibitors of programmed cell death 1 or its ligand, may be effective in treating metastatic HCC across various stages, not only in advanced cases.

## Conclusions

Metastasis of HCC to the PPS is rare. However, it is crucial to be aware of the potential for tumor metastasis to this region, particularly when bone destruction is observed. If a patient has known risk factors for HCC, clinicians must consider the possibility of metastatic HCC. FNAB is appropriate for diagnosing metastatic tumors in the PPS. The combination of locoregional and systemic therapies can effectively prolong the survival of patients with multiple metastases.

## Funding

The author(s) disclosed receipt of the following financial support for the research, authorship, and/or publication of this article: This study was supported by research grants from the National Natural Science Foundations of China (nº 82201301).

## Conflicts of interest

The authors declare no conflicts of interest.
